# Spotlights on ubiquitin-specific protease 12 (USP12) in diseases: from multifaceted roles to pathophysiological mechanisms

**DOI:** 10.1186/s12967-023-04540-6

**Published:** 2023-09-26

**Authors:** Kaiyi Niu, Yanlong Shi, Qingpeng Lv, Yizhu Wang, Jiping Chen, Wenning Zhang, Kung Feng, Yewei Zhang

**Affiliations:** Hepato-Pancreato-Biliary Center, The Second Affiliated Hospital of Nanjing Medical University, Nanjing, 210003 Jiangsu Province China

**Keywords:** USP12, Deubiquitination, Tumorigenesis, Immune response, Cancer

## Abstract

Ubiquitination is one of the most significant post-translational modifications that regulate almost all physiological processes like cell proliferation, autophagy, apoptosis, and cell cycle progression. Contrary to ubiquitination, deubiquitination removes ubiquitin from targeted protein to maintain its stability and thus regulate cellular homeostasis. Ubiquitin-Specific Protease 12 (USP12) belongs to the biggest family of deubiquitinases named ubiquitin-specific proteases and has been reported to be correlated with various pathophysiological processes. In this review, we initially introduce the structure and biological functions of USP12 briefly and summarize multiple substrates of USP12 as well as the underlying mechanisms. Moreover, we discuss the influence of USP12 on tumorigenesis, tumor immune microenvironment (TME), disease, and related signaling pathways. This study also provides updated information on the roles and functions of USP12 in different types of cancers and other diseases, including prostate cancer, breast cancer, lung cancer, liver cancer, cardiac hypertrophy, multiple myeloma, and Huntington's disease. Generally, this review sums up the research advances of USP12 and discusses its potential clinical application value which deserves more exploration in the future.

## Introduction

**Fig. 1:**
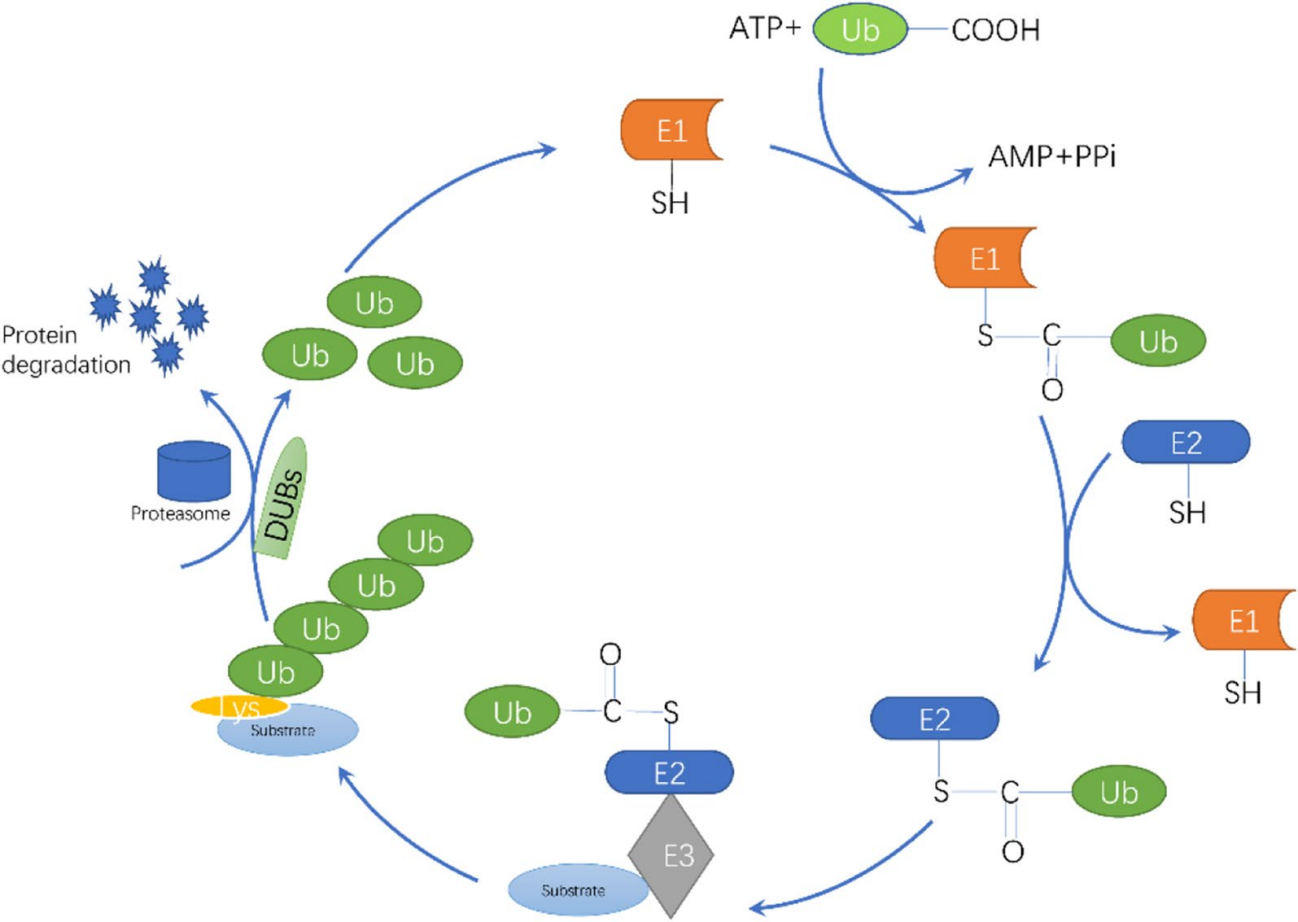
The ubiquitination and deubiquitination cycle. The ubiquitination process is conducted by three key enzymes that function sequentially. The ubiquitin-activating enzyme E1 promotes the formation of a thioester bond between the C-terminal carboxyl group of ubiquitin and the E1 cysteine sulfhydryl group in an ATP-dependent manner. Then ubiquitin is transferred from E1 to the active site of conjugating enzyme E2. Finally, the E3 ubiquitin ligase catalyzes the attachment of ubiquitin to the substrate through an isopeptide bond between the lysine of the target protein and the glycine of ubiquitin. The E3 enzymes are central in this cycle due to their ability to determine the specificity of the ubiquitination process via discriminating various substrates in cells. On the contrary, the deubiquitinases (DUBs) release the ubiquitin linked with substrates to regulate the stability and function of targeted proteins and the ubiquitin can be utilized in circulation

Ubiquitination is an essential type of post-translational modification performed by a small molecular protein containing 76 amino acid residues named ubiquitin, which is commonly expressed in eukaryotic cells and originally identified as a trigger for protein degradation by the 26S proteasome [1]. The core of ubiquitination modification is the formation of a stable isopeptide bond between the C-terminus of ubiquitin and the lysine residues of the target protein, which is performed by ubiquitin-activating enzyme (E1), ubiquitin coupling enzyme (E2), and ubiquitin ligase (E3) sequentially [2] (Fig. 1). Besides, polyubiquitination is also associated with E4 ligases, which engage in the formation of the E3–E4 or E4-substrate complex and coordinate the transfer of ubiquitin from E2 to the substrate [3]. The E3 enzymes play a central role in ubiquitination due to their capacity to determine the specificity of substrates in relevant ubiquitination processes [4]. A large number of studies have described the role of ubiquitination in cell physiological processes. On the one hand, it mainly regulates the proteasomal degradation of proteins [5]. On the other hand, ubiquitination has numerous nonproteolytic functions. Previous research reported that selective autophagy, which was essential for sustaining cellular homeostasis, was considered associated with ubiquitination. As an example, α-synuclein could be degraded by both proteasomal and autophagic after CHIP ubiquitination [6]. Moreover, histone H2B monoubiquitination occurs at Lys120 was found to be required for DNA double-strand break (DSB) repair in mammals [7]. Phospholamban (PLN) plays an important role in cardiac contractility by inhibiting sarcoplasmic reticulum Ca2 + ATPase, and it was found as a novel ubiquitination substrate of the von Hippel-Lindau protein (pVHL) E3 complex [8].

Similar to many post-translational modifications, ubiquitination is a reversible process catalyzed by deubiquitinases (DUBs) [9]. The deubiquitination catalyzed by DUBs possesses various functions to maintain cellular homeostasis. For example, dubiquitination can regulates many signaling pathways to ensure normal cell metabolism, including Hippo signaling, PI3K-AKT-mTOR signaling, and Wnt signaling, etc. [10–12]. Some immune checkpoint pathways were also reported to be regulated by deubiquitination, such as CTLA4/B7, LAG3 and TIGIT, etc. [13]. Other aspects of DUBs’ functions like DNA damage response, DNA repair (USP1/USP11), stem cell renewal (USP16/USP22) and so on have been researched too [14–16]. Hundreds of DUBs have been identified so far. Based on the characteristics of their different catalytic domains, DUBs are commonly classified into five major families, including ubiquitin-specific proteases (USPs), ovarian tumor-related proteases (OTUs), ubiquitin C-terminal hydrolases (UCHs), Machado-Joseph disease protein domain proteases (MJDs) and JAB1/MPN/MOV34 motif proteases (JAMMs). Among them, USPs is the largest family that contains about 58 members [17], and all other four families are cysteine proteases except for JAMMs which are metalloproteinases [18]. In recent years, researchers found that motifs interacting with ubiquitin-containing novel DUB family (MINDY), Zinc finger with UFM1-specific peptidase domain protein (ZUFSP), and monocyte chemotactic protein-induced protein family (MCPIP) also belong to the DUBs [19–21].

USPs participate in numerous physiological activities, such as cell proliferation, cell cycle, signaling pathways, inflammation, metabolism, and immunity [22–25]. The imbalance between ubiquitination and deubiquitination can lead to plenty of physiological disorders, including viral infection, inflammation, and cancer [26–28]. For instance, previous studies have shown that USP1 plays a critical role in colorectal cancer, and its knockdown can induce cell cycle arrest in the G2/M phase [29]. Also, dual regulation of FBW7 by USP28 can act as a tumor promoter or suppressor, depending on the autocatalytic ubiquitination status of FBW7 [30, 31]. USP21 promotes pancreatic cancer cell stemness by deubiquitinating and stabilizing the TCF/LEF transcription factor TCF7 [32]. Although the studies of DUBs have made tremendous achievements over the past decades, its mechanisms still need deeper investigation and there exists no systematic review of USP12 currently. In this review, we focus on the specific role of USP12 in tumor progression and immune response to obtain new insight into the mechanisms of USP12.

### The structure and physiological functions of USP12

**Fig. 2:**
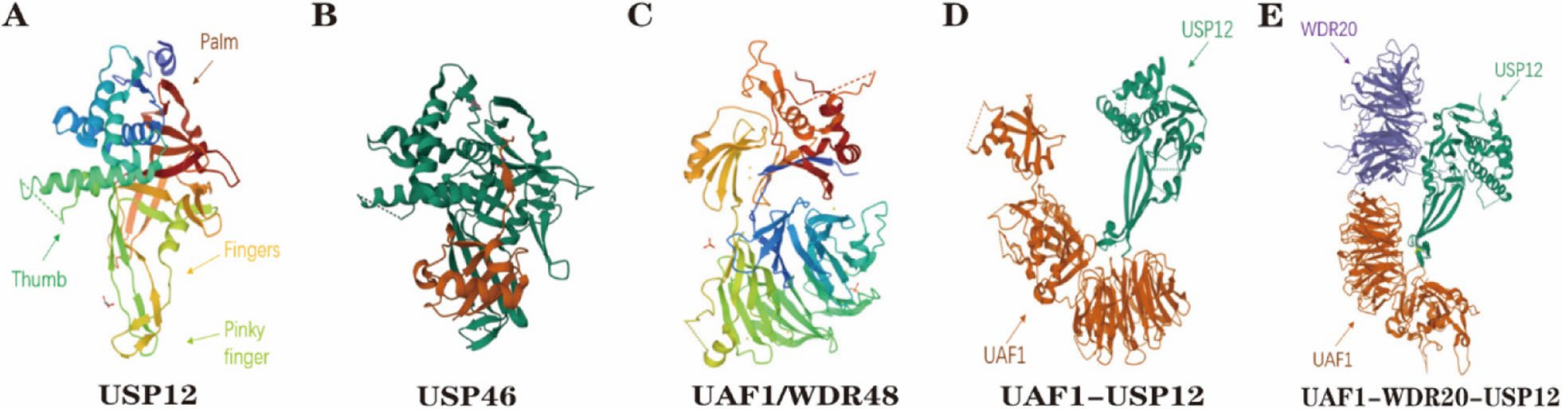
The structure of USP12 and related complexes. **A** The crystal structure of free USP12 with Thumb (cyan), Palm (brown), Fingers (yellow), and Pinky Finger (green). The Pinky Finger is separated from other finger domains and possesses a kind of coiled structure that may endow USP12 with more plasticity to exert its functions. **B** The crystal structure of free USP46 has a high similarity with USP12. **C** The crystal structure of free UAF1/WDR48.** D** The structure of the UAF1-USP12 complex. **E** The structure of UAF1-WDR20-USP12 complex

As demonstrated in previous studies, many USPs have modular structures that contain not only catalytic structural domains but also additional protein–protein interaction and localization structural domains [33]. The core catalytic structure of USPs consists of three sub-structural domains, the finger, palm, and thumb domains. The ubiquitin core is held by the "finger" and is responsible for supporting the globular domain of ubiquitin, while the catalytic center is located between the "palm" and "thumb" subdomains [34]. The catalytic core domain of USPs contains a conserved cysteine catalytic triad, and the extended finger domains together with the palm and thumb domains form the binding pocket for ubiquitin, recognizing the extended ubiquitin tail and presenting its C-terminus to the active site cysteine [35]. USP12, localized at chromosome 13q12.13, is a small-molecule protein that contains 370 amino acid residues and possesses high sequence similarity and conserved catalytic structural domains with USP46 and USP1 [36]. USP1, USP12, and USP46 constitute a subfamily of USPs which contain a single USP domain and share a common WDR partner WDR48 (also named USP1-associated factor 1; UAF-1) whose binding can stimulate the activity of these USPs [37]. The overall structure of the USP46-WDR48-WDR20 complex is very similar to that of the USP12-WDR48-WDR20 complex. Besides, USP12 and USP46 can also bind with WDR20 but USP1 cannot; and the activity of USP12 and USP46 can be activated by WDR48 and WDR20 independently and synergistically [38, 39]. However, unlike the general USPs, the outer edge of the finger domain of USP12 has a unique curly structural sequence called Pinky Finger that is separated from the rest of the finger domains, which shows the unusual structural flexibility of the finger structural domains of this enzyme [40]. DUBs are ubiquitous and can present in almost all cell compartments [41]. For USP12, its subcellular localization still has controversy due to some studies describe it as predominantly cytoplasmic or nuclear protein [26, 42–44]. The specific subcellular localization of USP12 can be regulated flexibly by some other proteins. For instance, E1 enzyme was reported to relocalize USP12 from the cytoplasm to the nucleus, and could recruit USP12 to the viral origin in a UAF1-dependent manner in HPV DNA replication [26]. Usp12 could translocate from the nucleus to the cytosol on TCR stimulation, and this relocalization process requires one or more kinases like phosphatase enzymes [45]. Moreover, WDR20 plays a crucial role in modulating the USP12-UAF1-WDR20 complex shuttling between the plasma membrane, cytoplasm, and nucleus [46]. Thus, USP12 can be expressed in different cell compartments and regulated complexly and precisely (Fig. 2).

USP12 has a variety of functions and properties. The combination of USP12 with USP46 deubiquitinated nucleosomal histones H2A and H2B, firstly verifying the regulatory role of this protease in cleaving ubiquitin from proteins [44]. USP12 has also been reported to be associated with DNA damage repair [47, 48]. In addition, USP1, USP12, and USP46 play important roles in anogenital HPV DNA replication through association with UAF1 and E1 [26]. USP12 also promotes LPS-induced signaling in macrophages through the dephosphorylation of IκBα [49]. In the aspect of cell biogenesis, USP12 promotes cell cycle progression by upregulating the transcription of BMI-1, c-Myc, and cell cycle protein D2 [50]. Dysregulation of USP12 can induce a variety of diseases. For example, USP12 overexpression could exacerbate Ang II-induced cardiac hypertrophy by raising the METTL3 level [51]. Although various physiological functions of USP12 have been reported, its mechanisms in regulating biological activities and diseases still need to be further elucidated (Table 1).

**Table 1:** The underlying mechanisms and effects of USP12-substrates interactions

Substrate	Mechanism	Impact	References
PHLPP1	USP12 deubiquitunates and stabilizes PHLPP1, which is an negative regulator of PI3K-Akt pathway, and this effect depends on the interaction between PHLPP1 and the specific domain of WDR48 that between its phosphatase domain and PDZ domain	Induce tumor cell apoptosis and inbibit proliferation	[63]
PHLPPL/PHLPP	USP12 deubiquitinates and stabilizes PHLPPL/PHLPP, which can decrease pAkt level through dephosphorylating it at S213/S791 site to suppress PI3K/Akt signaling, and then increase AR stability to promote prostate cancer	Deplete Usp12 resulted in the sensitisation of PC cells to Akt inhibition irrespectively of AR status	[107]
PPM1B	USP12 stabilizes PPM1B via deubiquitinaion, which is considered as an inhibitor of NF-κB pathway by inactivating IKKβ, thus inhibiting NF-κB pathway to negatively regulate cell chemokine expression	Represses NF-κB signaling activity to regulate the production of chemokine like CXCL8, CXCL1 or CCL2	[66]
MDK	USP12 mediates MDK deubiquitination by cleaving K48-linked polyubiquitin chains, and then MDK can promote VEGF expression via Akt-mTOR pathway to promote breast cancer angiogenesis and tumorigenesis	Stabilize MDK to activate the Akt-mTOR pathway to promote BC angiogenesis and metastasis	[73]
BMI-1/c-Myc/cyclin-D2	USP12 upregulates the transcription of BMI-1, c-Myc and cyclin D2 to promote cell cycle progression, and L153S and R237C mutations significantly increased the USP12 deubiquitinating enzyme activity	Regulate HeLa cell cycle progression positively. Depletion of USP12 impaired cell growth by inhibiting cell cycle progression without causing apoptosis in HeLa cells	[50]
AR	USP12 stabilizes AR and increases the transcript levels of AR and AR-regulated genes PSA and TMPRSS2 through deubiquitinaion	Promote the proliferation and cell cycle progression of PC, and inhibit PC cells apoptosis	[42]
Bax	USP12 regulates the K63-linked polyubiquitin chain of Bax through deubiquitinating enzyme activity, and the Bax K189R mutant form has higher ubiquitination level and shorter half-life	Stabilize Bax to promote apoptosis	[69]
HMGB1	USP12 deubiquitinates and stabilizes HMGB1, thus inducing autophagy in multiple myeloma	Promote growth and survival of MM cells, and enhance bortezomib resistance in MM	[67]
mHTT	Usp12 suppress mHTT neuronal toxicity withnot requiring its deubiquitinating activity, suggesting that the functional specificity of Usp12 in suppressing mHTT toxicity is conferred by sequences that reside outside the conserved catalytic domains	Protect neurons through rescuing mHTT-mediated neurodegeneration	[70]
MMP14	Circ-ADRM1 recruits USP12 to impede the ubiquitination of MMP14 protein, thereby enhancing the stability of MMP14 protein	Promotes LUAD cell proliferation, invasion and migration through upregulating MMP14	[74]
IκBα	USP12 promotes NF-κB signalling in macrophages through the dephosphorylation of IκBα, which is a inhibitor of NFκB nuclear translocation	Promote LPS induced macrophage responses through the inhibition of IκBα	[49]
LAT/Trat1	Usp12 stabilizes LAT and Trat through deubiquitylating and preventing their lysosomal degradation	Maintain the proximal TCR complex for the duration of signaling	[45]
NLRP3	UAF1-USP12 complexes interact with p65 and inhibit its ubiquitination and degradation, thus promoting NF-κB activation, resulting in the enhancements of NLRP3	Facilitate NLRP3 inflammasome activation via targeting NLRP3 and p65	[99]
STAT1	USP12 translocates from the cytoplasm to the nucleus and then blocks CBP-induced acetylation of phosphorylated STAT1 (p-STAT1)	Maintain nuclear p-STAT1 levels and IFN antiviral efficacy	[102]

### The role of USP12 in tumorigenesis

Numerous articles have revealed the varying effects of DUBs on tumorigenesis. For instance, OTUD6B depletion also enhances cell migration and HIF-2α levels in a pVHL (Von Hippel-Lindau protein with missense point mutations)-dependent manner in clear cell renal cell carcinoma (ccRCC) [52]. While in Hepatocellular carcinoma (HCC), OTUD6B decreases HIF-1α accumulation in HCC cells under hypoxia via directly interacting with pVHL to reduce its ubiquitination and proteasomal degradation, thereby inhibiting HCC cell metastasis [53]. In addition to OTUD6B, deubiquitinating enzymes such as USP7, USP3, MINDY1, PSMD14, USP25, USP37, USP30, USP8, and USP33 have also been reported to affect phenotypes like cell proliferation, cell cycle, metastasis, apoptosis, and autophagy through various pathways [54–62]. Several studies reported the effects of USP12 on cell phenotypes, and we give a summary of the roles and mechanisms of USP12 in tumorigenesis in the following part.

The overexpression of USP12 in human colorectal cancer cells was previously found to inhibit cell proliferation, and siUSP12 could reverse this effect [63]. On the other hand, USP12 promoted prostate cancer (PC) cells proliferation by forming a complex with Uaf-1 and WDR20 and deubiquitinating AR, thereby increasing AR stability and transcriptional activity, whereas USP12 silencing led to a significant decrease in PC cell proliferation [42, 64]. Similarly, the positive or negative effect of USP12 on cell proliferation was also found in HPV-negative human cervical cancer, hepatocellular carcinoma (HCC), non-small cell lung cancer (NSCLC), and multiple myeloma (MM) [26, 65–67]. The diverse regulation of cell proliferation and cell cycle by USP12 indicates its functional diversity and deserves further study.

USP12 silencing could induce the upregulation of Bax in prostate cancer by regulating the TP53 signaling pathway [68], and in Hela cells, Bax could bind to USP12 in the nucleus [50]. The polyubiquitin chains shaped in Bax are located at k48 and k63, while USP12-mediated deubiquitination acts at the k63 but not the k48 site. Interestingly, although USP12 has deubiquitinating enzymatic activity towards Bax, it cannot regulate its protein expression level [69]. The mechanisms underlying the influence of USP12 on apoptosis have also been researched in some other diseases, such as cardiac hypertrophy, prostate cancer, and hepatocellular carcinoma [63, 64, 68]. In the context of autophagy, the study of multiple myeloma (MM) showed that USP12 could interact with the key autophagy mediator HMGB1 (high mobility group box-1) protein to deubiquitinate and stabilize it [67]. In Huntington's disease (HD), USP12 was identified as a potent inducer of neuronal autophagy due to its interaction with HD mutant protein (mHTT) [70–72]. Therefore, the mechanism of action of USP12 on apoptosis and autophagy merits more investigation to develop novel insight into therapeutic strategies.

Besides the above, USP12 has also been proven to mediate cancer invasion and metastasis. Human umbilical vein endothelial cell (HUVEC) migration and angiogenesis assays demonstrated that overexpression of USP12 could promote breast metastasis, and USP12 depletion undermined the formation of lung metastatic nodules [73]. Furthermore, Matrix metalloproteinase 14 (MMP14), a target gene of miR-1287-5p, promotes the proliferation, invasion, and migration of Lung adenocarcinoma (LUAD) cells. Circ-ADRM1 recruited USP12 to block the ubiquitination of MMP14 protein, thereby enhancing the stability of MMP14 protein [74]. Hence, USP12 is associated with tumor invasion and metastasis and has the potential to be a therapeutic target against tumor metastasis.

### USP12 and immunity

USPs have been shown to make a difference in the development of tumor immunogenesis. For example, USP15-deficient T cells have been demonstrated to induce PD-L1 and CXCL2 expression through the production of IFN-γ and facilitate infiltration of T-bet + regulatory T cells and myeloid-derived suppressor cells (MDSCs), thus leading to tumor progression [75]. Knockdown of USP14 decreases indoleamine 2,3 dioxygenase 1 (IDO1) expression, reverses cytotoxic T-cell suppression, and increases responsiveness to anti-PD-1 therapy [76]. USP1, in turn, promotes Th17 cell differentiation and attenuates Treg cell differentiation, thereby promoting the development of inflammatory diseases [77]. Similar to the USPs mentioned above, USP12 can also exert immunomodulatory effects through various mechanisms (Table 2).

**Table 2:** The regulation of USP12 and immunity-related pathophysiology

Pathophysiology	Target	Mechanism	Effect	References
T cells	LAT/Trat1	Deubiquitinate and stabilize LAT and Trat1	Stabilize TCR complex on the T cell surface during signaling	[45]
Macrophages	IkBα/Sp1	Activate NF-κB pathway via regulating IκBα	Affect LPS-induced macrophages response	[49]
Inflammation	NLRP3	Promote NF-κB to enhance NLRP3 inflammatory microsome activation	Promote NLRP3-mediated inflammatory response	[98, 99]
Viral infection	STAT1	Regulate various cytokines like TNF, IFN, IL	Inhibit viral infection	[102]

### The effect of USP12 on the regulation of PD-1 and TME

Studies have illustrated that USP12 was associated with many aspects of TME, including immune checkpoints, chemokines, immune cells and viral infection [78–80]. In terms of NSCLC, USP12 was decreased upon the activation of AKT-mTOR signaling, and USP12 could reduce the levels of several chemokines, including CXCL8, CXCL1, CCL2, and CCL5, which were relevant to immune cell recruitment. This effect was mainly due to USP12's inhibitory effect on NF-κB signaling activity by deubiquitinating phosphatase PPM1B, which regulated the chemokine secretion [81]. Downregulation of USP12 contributed to the development of immune suppressive TME in NSCLC. The FACS analysis of immune cell profiles showed that the expression of USP12 was negatively correlated with tumor-associated macrophages (TAMs) and PD-L1 (CD274), which were significant for tumor immune therapies [82, 83]. Given the influence of USP12 on PD-L1 expression, researchers also tested the impact of USP12 inhibitors on anti-PD-L1 therapy and the results showed that USP12 silencing desensitized tumors to anti-PD-L1 treatment. Interestingly, the expression of chemokines CXCL1 and CCL2 largely counteracted the inhibitory effect of USP12 on PD-L1 expression as well as CD31 + cells, and effectively attenuated the activation of CD8 + and CD4 + T cells. In addition, a trend toward decreased expression of CD163 [84, 85], which was considered as a biomarker of M2 macrophages, was observed in the condition of low USP12 expression. After USP12 was inhibited, CD206-expressing Bone marrow-derived macrophages (BMDMs) were also remarkably increased. The above results suggest that USP12 plays an important role in the development of tumor immunosuppressive TME [66].

Additionally, USP12 affected colorectal tumor growth by intrinsically regulating IFN-γ expression in CD4 + T cells but not CD8 + T cells. USP12 deficiency promoted the expression of inducible nitric oxide synthase (iNOS) in MDSCs by inhibiting the p65-NF-κB signaling pathway, and MDSCs with high iNOS expression inhibit T-cell function, resulting in poorer tumor response to chemotherapy [86]. Also, USP12 negatively regulated the expression of PD-L1 on MDSCs in colorectal cancer. MDSCs with high PD-L1 expression can interact with PD-1 on T cells and induce T cell apoptosis [87]. These results indicate that USP12 is a potential therapeutic target and may contribute to enhanced anti-tumor immunotherapeutic effects [78].

### *USP12 and CD4* + *T cells*

USP12 has been reported to be one of the key regulators of CD4 + T cells. USP12 modulates the phenotype of CD4 + T cells in terms of differentiation, activation, and proliferation, but not in CD8 + cells. Studies showed that the expression of IFN-γ, TNF-α, IL-2, IL-17A, CD69, and CD44 was reduced in CD4 + T cells in USP12 knockout cells, while CD62L was significantly upregulated. IL-2 is a key regulator of CD4 + T cell proliferation, and CD4 + T cells exhibit reduced activation and proliferation in the presence of USP12 deletion. However, USP12 deficiency does not affect the proportion of Th2 and Treg cells in CD4 + T cells and the intracellular expression of IFN-γ and TNF-α in CD8 + T cells, nor does it affect the activation of CD8 + T cells. Further studies reveal that USP12 stabilizes BCL-10 by deubiquitination and activates the NF-κB signaling pathway, thereby activating CD4 + T cell response. Moreover, the immune response of USP12 knockout mice to L. monocytogenes infection was significantly reduced [79].

### The regulation of TCR by USP12

T cell surface receptors (TCRs) are activated upon binding to antigens thus initiating the immune response through a series of signaling, which can be modulated by phosphorylation and ubiquitination. DUBs that are known to participate in TCR signaling include OTUB1, USP34, and USP9X [88–92]. High ubiquitination of LAT has been reported to reduce TCR levels [93], whereas overexpression of Trat1 stabilizes TCR [94]. An investigation showed that in USP12-deficient cells, both LAT and Trat1 were degraded by ubiquitination and thereby downregulating TCR expression. This process could be reversed by USP12. After stimulating the TCR with an anti-CD3 antibody, phosphorylation of the USP12 cytoplasmic pool could be induced, allowing USP12 to translocate from the nucleus to the cytoplasm and acted directly on the substrate proteins LAT and Trat1 to stabilize the TCR complex on the T cell surface during signaling. Upon stimulation of USP12 knockdown T cells, LAT phosphorylation was defective and exhibited diminished NFκB, NFAT, and MAPK activity in Jurkat cells, the activity of these molecules could be rescued by USP12 expression reconstitution. USP12 removes ubiquitin chain modifications mediated by E3 ligase and its activity may be regulated by TCR signaling [45]. Interestingly, Cbl-b and Itch were isolated among the E3 ligases interacting with USP12. GRAIL is another E3 ligase that has been shown to ubiquitinate CD3. GRAIL and Cbl-b deficiency induce TCR stabilization as well as enhance T cell responses [95–97]. However, whether USP12 counteracts the activity of GRAIL, Cbl-b, Itch or other E3 ligases remains to be further investigated.

### The association of USP12 and macrophage

The role of USP12 in macrophages has also been explored. USP12 could regulate the LPS-induced pro-inflammatory response in macrophages and was required for LPS-mediated macrophage activation via the NF-κB pathway. Knockdown of USP12 inhibited the NF-κB pathway by reducing the phosphorylation level of IkBa (degraded form), which was a kind of inhibitor of NF-κB nuclear translocation in LPS-induced macrophages, and an increased number of dephosphorylated IkBa was then translocated to the nucleus to inhibit the NF-κB pathway. Moreover, the knockdown of USP12 increased the total protein level of IkBa in macrophages. USP12 levels were upregulated in macrophages for 12 h after the treatment of LPS, but downregulated after 24 h. This may be related to the fluctuating expression of the transcription factor Sp1. The USP12 mRNA levels in macrophages were enhanced by overexpression of Sp1, and the downregulation of USP12 in 24 h may be related to Sp1 depletion. USP12 was required for the induction of NF-κB-dependent proinflammatory factors iNOS and IL-6. Knockdown of USP12 significantly reduced LPS-stimulated iNOS protein levels, an effect that is probably limited by LPS stimulation itself, as knockdown of USP12 did not alter IFN-γ-induced iNOS levels. In addition, the knockdown of USP12 inhibited LPS-induced IL-6 synthesis and phosphorylation of ERK1/2 and p38-MAPKs but did not attenuate IFN-β production. Thus, USP12 is an LPS-sensitive DUB in macrophages that can affect LPS-induced macrophage signaling pathways by regulating IkBa phosphorylation, suggesting the importance of inhibiting USP12 in controlling macrophage hyperactivation [49].

### USP12 and inflammation

In inflammatory response, NOD-like receptor protein 3 (NLRP3) can detect microbial infections, and then activate NLRP3 inflammatory microsome, thus influencing the development of inflammation. Deubiquitination of NLRP3 is considered to be a critical step for NLRP3 inflammatory microsome activation, and NF-κB is the key factor for NLRP3 expression [98]. The UAF1-USP12 complex was found able to enhance NLRP3 and inflammatory factor (including IL-1β, TNF, and IL-6) production by inhibiting p65 ubiquitination degradation and promoting NF-κB activation. Downregulation of USP12 notably suppressed NLRP3 expression in unstimulated and LPS-stimulated macrophages. Researchers found that ML323, which is a novel NLRP3 inflammatory microsome inhibitor, can specifically inhibit the UAF1/USP1 but not the UAF1-USP12 and UAF1-USP46 complexes [36, 99].

### USP12 and virus infection

Besides the above studies, in virus infection, EBV nucleoproteins EBNA3A, EBNA3B, and EBNA3C were found to be highly associated with the USP46-USP12 complex, and EBNA3A and EBNA3C are essential for EBV-mediated transformation of resting B lymphocytes into immortalized lymphoblastoid cell lines (LCL) [100]. USP12 interacted with viral capsid protein (CP) in Epinephelus coioides to inhibit viral infection and positively regulated the levels of associated inflammatory factors, including TNF-α, IL-1β, IL-6, IL-8, IRF, and IRF7 [101]. Furthermore, USP12 orthogonally regulated interferon (IFN) anti-viral signaling without its deubiquitination activity. In IFN signaling, USP12 was found to translocate from the cytoplasm to the nucleus and then blocked CREB-binding protein (CBP)-induced acetylation of phosphorylated STAT1 (p-STAT1), thereby maintaining nuclear p-STAT1 levels and IFN anti-viral efficacy [102]. A recent study found that USP12 could stabilize interferon-γ inducible protein 16 (IFI16) by regulating its k48-linked ubiquitination and then promoted IFI16-STING-IRF3 and p65-mediated antiviral signaling, which made the host more resistant to DNA virus but not RNA virus [80].

Overall, the modulatory functions of USP12 in the immune system are complicated. The regulation of a certain pathway or molecule in different cells may have opposite effects. For example, USP12 is described above as promoting NF-κB activation for NLRP3 inflammatory microsome production, but in NSCLC it inhibits NF-κB signaling activity and regulates chemokine secretion by deubiquitinating and stabilizing PPM1B. Apart from that, USP12 can translocate from the cytoplasm to the nucleus during signaling to function in maintaining nuclear p-STAT1 levels and IFN antiviral efficacy. However, in the TCR-related studies, anti-CD3 stimulation of the TCR translocated USP12 from the nucleus to the cytoplasm to act on the substrate proteins LAT and Trat1 to stabilize the TCR. The different translocation patterns of USP12 in different signaling pathways show that it is dynamically regulated in intracellular compartments to adapt to various cellular signaling. The diverse regulation of immune cells and molecules by USP12 may be related to the specificity of cells or substrates and influenced by the binding of USP12 cofactor UAF-1 or WDR20. Compared to the DUBs mentioned above, USP12 has been more extensively studied in the aspects of immunity, inflammation and antiviral with adequate experimental support, including effects on various immune cells, cytokines, and viral infections. Therefore, USP12 has great potential to be a therapeutic target for diseases associated with immune microenvironment alterations, and the mechanisms of USP12 in the immune system deserve deeper investigation.

### USP12 and signaling pathways

#### USP12 and Notch signaling pathway

The NOTCH pathway is a conserved signaling pathway conducted by binding to receptors on transmembrane ligands and adjacent cells which plays an important role in cell proliferation or differentiation, and its dysregulation can cause a variety of diseases [103]. Ubiquitination has an important impact on NOTCH. Studies have shown that USP12 is a negative regulator of NOTCH. USP12 deficiency can lead to increased expression of inactive NOTCH receptors on the cell membrane, which in turn promotes NOTCH signaling activation. USP12 has a specific deubiquitination effect on inactive NOTCH, which is necessary for the degradation of Notch in late endosomes/lysosomes. A study of enzymatic properties of the USP family suggests that the USP12-UAF1 complex is able to cleave all types of chains (except linear) and could theoretically hydrolyze the Lys-29-linked chains formed on Notch by virtue of Itch/AIP4 E3 ubiquitin ligase activity [104–106]. However, more studies are needed to confirm the relationship between the regulation of NOTCH signaling pathway by USP12 and the development of the disease.

#### USP12 and NF-κB signaling pathway

The interaction of USP12 with NF-κB signaling is mainly related to the regulation of the immune system. USP12 can inhibit NF-κB signaling activity to regulate the production of chemokines (such as CXCL8 and CXCL1), which then modulate the tumor immune microenvironment to alter the response of NSCLC to immunotherapy [66]. In addition, USP12 activates CD4 + T cell proliferation by stabilizing BCL10 and targeting the NF-κB signaling pathway. However, USP12 does not have this effect in CD8 + T cells [79]. In the inflammatory response, the UAF1-USP12 complex removes K48-linked ubiquitination of the NF-κB subunit p65 and enhances its expression, activating NF-κB signaling and thus promoting NLRP3 transcription. NLRP3 inflammasome plays an important role in host defense and contributes to the pathogenesis of inflammatory diseases [99]. Moreover, USP12 is required for LPS-mediated macrophage activation, possibly by activating the NF-κB pathway [49]. Therefore, the study of USP12 in NF-κB signaling contributes to the in-depth understanding of the tumor immune microenvironment, providing new insights into immunotherapy.

#### USP12 and PI3K-Akt-mTOR signaling pathway

Gene set enrichment analysis confirms an inverse correlation between transcription levels of USP12 and Akt-mTOR activation in NSCLC, but USP12 overexpression has no significant effect on Akt phosphorylation. In addition, blocking Akt-mTOR signaling with selective Akt inhibitor API-2 or mTOR inhibitor rapamycin significantly increased USP12 expression. These data suggest that USP12 is related to the carcinogenic effect of AKT-mTOR signaling in NSCLC [66]. USP12-Uaf-1-WDR20 complex directly deubiquitinates and stabilizes PHLPP and PHLPPL, resulting in a decrease in the active p-Akt level. The reduced pAkt in turn downregulates AR Ser213 phosphorylation, thereby enhancing AR stability and transcriptional activity, promoting the development of prostate cancer [107]. USP12 can also activate the Akt signaling pathway in tumors and endothelial cells by upregulating MDK, and promoting VEGFR3 expression through the mTOR signaling pathway, which then promotes the occurrence and progression of breast cancer [73].

#### USP12 and MAPK signaling pathway

**Fig. 3:**
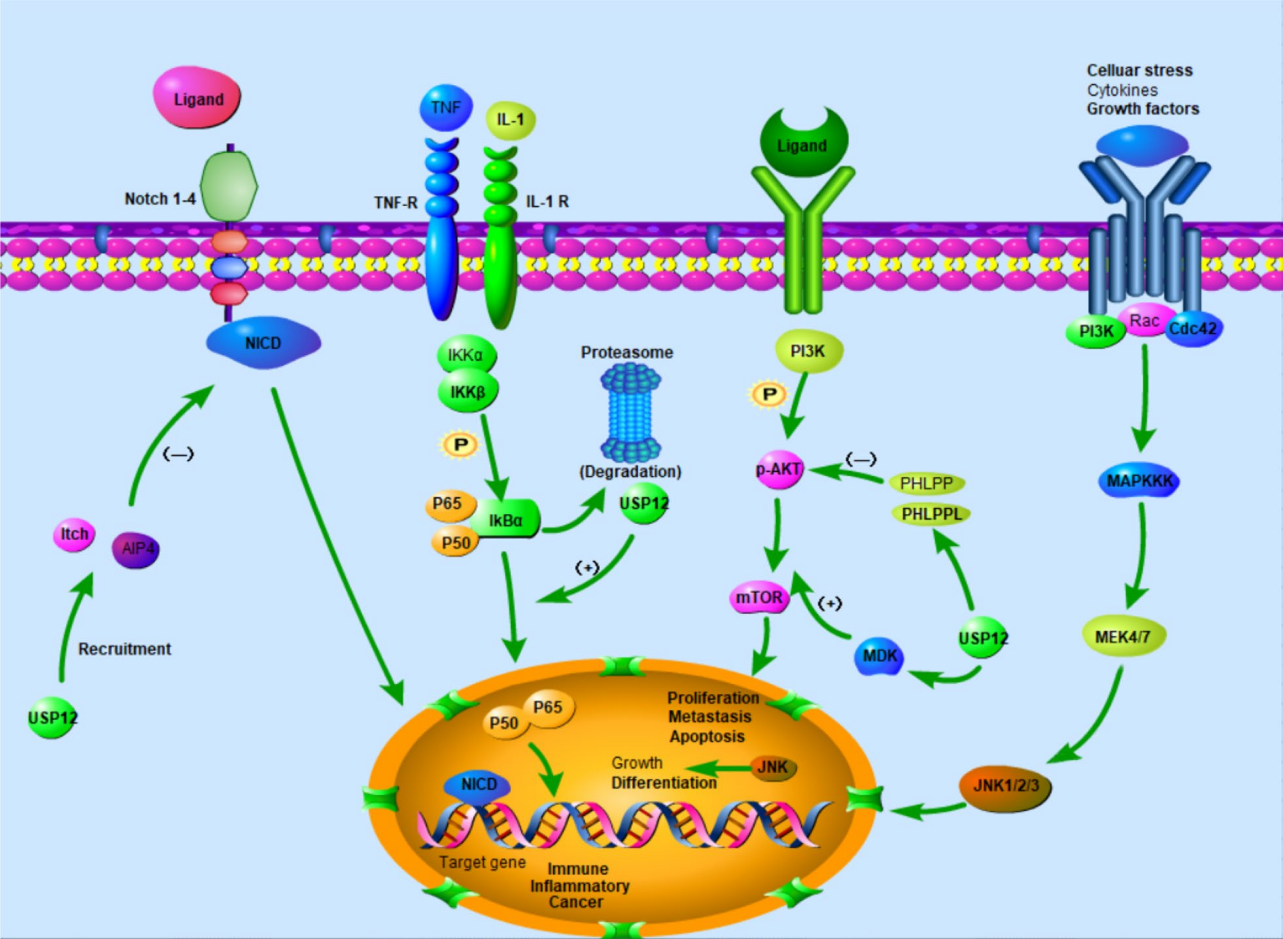
The USP12-related pathways

In HCC, phosphorylated p38 (p-p38) and phosphorylated JNK (p-JNK) are activated after USP12 knockout, and the apoptosis markers cleaved-caspase 9 and cleaved-caspase 3 are upregulated. This activation was reversed by p38/MAPK inhibitor SB202190. Therefore, USP12 can regulate the occurrence and development of HCC through the p38/MAPK pathway and the interaction between USP12 and the MAPK signaling pathway in other disease requires further study [65] (Fig. 3).

#### USP12 and disease

The role of DUBs in disease has been extensively reported. USP25 ameliorates myocardial hypertrophy through deubiquitination to maintain the stability of SERCA2a protein (an anti-hypertrophy protein) [108]. STAMBP from the JAMM family stabilizes RAI14 protein by inhibiting K48 ubiquitination of RAI14 and promoting the development of triple-negative breast cancer [109]. USP28 deubiquitinates and stabilizes FOXM1, which subsequently leads to the activation of the Wnt/β-linked protein pathway and promotes the progression of pancreatic cancer [110]. In addition, DUBs such as USP13, USP25, PSMD12, YOD1, PSMD7, USP47, OUTB1, and TRABID also exert promotional or inhibitory effects on a wide variety of cancers [111–118]. Similarly, USP12 has also been studied in tumors and identified as one of the 12 most frequently overexpressed cancer-related genes located near amplified super enhancers [119], and therapeutic approaches targeting USP12 deserve more attention. The following part summarizes the role of USP12 in human disease and the therapeutic advances to gain a novel understanding of the physiopathological mechanisms associated with USP12 (Table 3).

**Table 3:** The mechanism of USP12 in different diseases and related pathways

Disease	Interactor	Mechanism	Effect	References
Prostate cancer	AR	Increase AR expression and stability to promote PC via the PI3K-Akt pathway	Promote proliferation and cell cycle, inhibit apoptosis	[42, 107]
Breast cancer	MDK	Stabilize MDK to promote angiogenesis and metastasis	Promote VEGF expression and metastasis	[73]
Lung cancer	MMP14/PPM1B	Enhance PPM1B stability to inhibit NF-κB-dependent chemokine expression	Promote proliferation, migration and invasion, reshape TME	[66, 74]
Liver cancer	P38/JNK	Inhibit the p38-MAPK pathway to promote HCC progression	Promote proliferation and inhibit apoptosis	[65]
Multiple myeloma	HMGB1	Increase HMGB1 expression and induce HMGB1-mediated autophagy	Induce autophagy and drug resistance	[67]
Cardiac hypertrophy	p300	Inhibit p300 degradation and promote METTL3 transcription	Promote Ang II-induced cardiac hypertrophy	[51]
Huntington’s disease	mHTT	Induce autophagy and decrease mHTT-mediated toxicity	Alleviate mHTT-mediated toxicity and induce autophagy	[70]

### Prostate cancer

AR is pivotal in the development of prostate cancer (PC) [120]. It has been indicated that AR can be regulated by many ubiquitinated or deubiquitinated enzymes, such as MDM2, CHIP, NEDD4 [121–123], and USP26 [124]. USP12 binds to Uaf-1 and WDR20 to form a complex to deubiquitinate AR, thereby increasing AR protein stability and transcriptional activity, while the depletion of WDR20 and Uaf-1 displays the opposite effect. USP12-Uaf-1-WDR20 increases AR protein levels, but the knockdown of USP12 has no significant effect on AR transcript levels, suggesting that USP12 itself may not regulate AR gene expression. It has been mentioned previously that USP12 silencing inhibits PC cell proliferation, induces cell cycle arrest, and promotes apoptosis [42]. In addition, the USP1-Uaf-1-WDR20 complex directly deubiquitinates and stabilizes the Akt phosphatases PHLPP and PHLPPL, leading to a decrease in active pAkt levels, which then downregulates AR phosphorylation and enhances AR stability and transcriptional activity. This provides a therapeutic approach that targets the PC PI3K-Akt pathway. Researchers found that USP12-silenced PC cells were significantly more sensitive to Akt inhibitors and were independent of AR status [107]. ML323 is a USP1/Uaf-1 inhibitor [36], and given that UAF1 is an indispensable active regulatory cofactor of USP12, it is reasonable to assume that ML323 also has some inhibitory effect on PC development, which requires more experiments to testify. USP12 also deubiquitinates AR and MDM2, which in turn controls TP53 levels and exerts a regulatory effect on PC by controlling the TP53-MDM2-AR-AKT signaling pathway [68]. Moreover, Galeterone, a novel small molecule anti-androgen drug targeting USP12 and USP46, has been developed and is effective in both desmoplastic resistance and AR-negative prostate cancer [125]. The above findings suggest that USP12 plays an essential role in PC, showing its potential in anti-cancer therapeutic design, which deserves more in-depth studies.

### Breast cancer

USP12 is associated with breast cancer metastasis. USP12 binds and cleaves K48-linked polyubiquitin chains to mediate MDK deubiquitination, leading to the upregulation of MDK and promoting angiogenesis and metastasis. MDK activates the Akt signaling pathway in tumor vascular endothelial cells and promotes VEGF expression through the mTOR signaling pathway. Overexpression of USP12 promotes the secretion of VEGF through MDK and fosters the angiogenesis of human umbilical vein endothelial cells (HUVEC), while the knockdown of USP12 inhibits this effect. Moreover, the knockdown of USP12 reduces the ability of lung metastasis and CD31 (vascular endothelial cell marker) protein levels in mice with breast cancer. Also, high USP12 and MDK expression predicts poor prognosis in breast cancer patients. Thus, the USP12-MDK axis may be a potential target for breast cancer metastasis treatment [73]. PRR11 and SKA2 are potential oncogenes in breast cancer. The expression levels of PRR11 and SKA2 were found upregulated in breast cancer and could be negatively regulated by p53. Knockdown of PRR11 and SKA2 inhibited migration and invasion of breast cancer cells. Further studies showed that PRR11 and SKA2 could affect the expression of several genes related to cell migration and invasion when they have been silenced alone or in combination, including USP12. The USP12 expression was decreased when PRR11 and SKA2 were silenced, suggesting that there may be some regulatory mechanisms among them that need to be further explored [87]. However, whether USP12 is associated with P53 in breast cancer remains to be further investigated. The above studies suggest that targeted drugs against USP12 could help inhibit the development and metastasis of breast cancer, but the exact mechanism needs more experimental confirmation.

### Lung cancer

The effect of USP12 on lung cancer has also been reported. Both MMP14 mRNA and protein levels were significantly overexpressed in lung adenocarcinoma (LUAD) cells relative to normal tissue. MMP14 promotes proliferation, migration, and invasion in LUAD and is affected by deubiquitination. It was demonstrated that circ-ADRM1 recruited USP12 to promote the deubiquitination of MMP14 protein, thus enhancing the stability of MMP14 protein. Therefore, it is possible to inhibit the activity of MMP14 by targeting USP12, thus achieving a therapeutic effect on LUAD [74]. It has also been described that USP12 is downregulated in NSCLC and able to regulate tumor chemokine secretion or induce macrophage recruitment and decrease T cell activity to promote tumor microenvironment development. Downregulation of USP12 also promotes NSCLC angiogenesis and upregulates PD-1 thus inducing resistance to anti-PD-L1 therapy [66]. Moreover, recent research has shown that USP12 directly interacts with and deubiquitinates RRM2 in NSCLC. Knockdown of USP12 causes DNA replication stress and retards tumor growth in vivo and in vitro [126]. All these findings indicate the great potential of USP12 to be a therapeutic target in lung cancer and deserve more research to explore novel effective therapies.

### Hepatocellular carcinoma

USP12 has been researched in hepatocellular carcinoma to some extent. Relative to normal tissues, USP12 expression was elevated at both mRNA and protein levels in HCC tumor tissue samples. Knockdown of USP12 inhibited HCC cell proliferation and promoted apoptosis via the p38-MAPK pathway. Besides, USP12 could induce HCC cell cycle arrest at the G2/M phase via the cell cycle protein-dependent kinase 1/cyclinB1 axis. Interestingly, high USP12 expression may indicate poor differentiation of HCC and correlate with clinicopathological staging. This reveals that USP12 may be a target for HCC therapy, and further studies are needed [65].

### Cardiac hypertrophy

Pathological cardiac hypertrophy is a major risk factor for heart failure and the activation of angiotensin II (Ang-II) is central [127]. Methyltransferase-like 3 (METTL3) is the predominant enzyme catalyzing m6A deposition [128], and there is growing evidence showing that elevated METTL3 is detrimental to cardiovascular health. It has been demonstrated that the mRNA and protein expression levels of USP12 are elevated in Ang II-induced hypertrophic cardiac myocytes. USP12 inhibits the degradation of p300 by deubiquitinating its K48-linked polyubiquitination chains and stabilizes p300 protein, which in turn promotes METTL3 transcription via enhancing METTL3 promoter activity and eventually exacerbates Ang II-induced cardiac hypertrophy. These results reveal a potential method to treat myocardial hypertrophy by inhibiting USP12 and provide new insights into the treatment of heart failure [51].

### Multiple myeloma

Previous studies show that USP12 promotes the growth of multiple myeloma (MM) cells and USP12 knockdown significantly inhibits cell proliferation. Furthermore, USP12 induces MM cell autophagy via deubiquitinating and stabilizing HMGB1. After the knockdown of USP12, the autophagosomes labeled by LC3, which is a kind of autophagy marker, are significantly reduced in MM cells. Autophagy is a major factor contributing to BTZ resistance [129]. USP12 high expression promotes HMGB1 expression to induce autophagy and drug resistance in MM cells. In BTZ-resistant cell lines, LC3 levels as well as USP12 and HMGB1 levels are significantly elevated. In contrast, the knockdown of USP12 restores the sensitivity of drug-resistant cells to BTZ. The above results provide novel understandings of USP12-mediated regulation of MM autophagy and suggest potential avenues for targeted therapy [67].

### Huntington’s disease

Huntington's disease (HD) is a progressive neurodegenerative disorder attributed to CAG repeat expansion, which confers Mutant HTT (mHTT) toxic functions that interfere with immune and mitochondrial function, and is aberrantly modified post-translationally [130]. USP12 was identified as a potent inducer of neuronal autophagy in HD due to its interaction with mHTT. USP12 depletion in neurons exacerbated mHTT-mediated toxicity, and overexpression of human USP12 reduced the risk of death associated with mHTT but did not affect the survival of HTT-expressing neurons. Interestingly, this function of USP12 was not required for its catalytic activity as a deubiquitinating enzyme. It was found that the mutant USP12-C48S, which was produced by replacing the cysteine active site (C48) of USP12 with serine (C48S), still could rescue mHTT-mediated toxicity in neurons and was unaffected by the knockdown of USP12 cofactors UAF-1 and WDR20 that important for USP12 activity. This suggested that the function of USP12 in inhibiting mHTT toxicity was probably exerted by sequences located outside the catalytic center, which meant USP12 may have a unique non-catalytic function in HD. Notably, although autophagy is the major method of mHTT clearance, the mechanism of the effect of USP12 on mHTT autophagic clearance is still not understood. USP12 may only act on small subgroups of mHTT or indirectly affect mHTT by acting on other substrates Furthermore, USP46 shares approximately 90% sequence similarity with USP12 [35], but cannot rescue mHTT-mediated toxicity like USP12. Moreover, there has no difference in the efficiency of USP12 and non-catalytic mutant of USP12 in stimulating the clearance of LC3, which is an autophagy protein that incorporates into autophagosomes, and selectivity degraded via autophagy. This suggests that USP12 may reduce mHTT toxicity by exerting a non-deubiquitinating effect [70]. Shortly, these results identify USP12 as a potent modulator of autophagy and mHTT-mediated neurotoxicity in HD, providing a new idea for the treatment of HD.

Although numerous studies have been conducted on USP12, there still exists some limitations in the understanding of USP12's functions. On the one hand, most current researches have focused on the effect of USP12 on pathophysiological changes, the exploration of the USP12 inhibitors is still preliminary and needs more investigations. The role of USP12 in inflammation, immunity and antiviral has been described in some papers, but there is a lack of sufficient experimental support for the development of inhibitors against USP12 in the field of immunotherapy or antiviral therapy. More researches are needed to determine whether treatments targeting USP12 are effective in treating immune disorders and what advantages they have over conventional immunotherapeutic approaches, or whether they have synergistic effects in combination with conventional immunotherapy. On the other hand, whether the promotional or inhibitory effects exerted by USP12 in different diseases and immune responses require its deubiquitinating enzyme activity or the binding of the cofactor like USP46 or WDR48, and the specific mechanisms still need to be studied in greater depth. Galeterone was reported to be able to inhibit USP12 and USP46 enzymatic activity to control prostate cancer growth and survival, which is not surprising considering their high degree of homology and functional overlap [125]. Due to the high structural similarity of USP12, USP46, and USP1, the inhibitors of USP1 like ML323, pimozide [131], and SJB3-019A [132] may also be effective for USP12, and more studies are required to verify this hypothesis.

Besides, developing new drugs to treat cancer from scratch is a time-consuming, expensive and inefficient process due to the complexity of the mechanisms involved in cancer development. In recent years, drug repurposing has evolved as an effective alternative to the search for other indications for which drugs have already been approved by the United States Food and Drug Administration (FDA) and applied in other diseases, including cancer, autoimmune disease and viral infection etc., and multiple drug repurposing approaches have been testified with successful results [133–136]. Deubiquitination was also reported to be involved in drug repurposing. For example, the interaction between USP5 and oxysterol-binding protein-related protein 8 (ORP8) facilitated the accumulation of ORP8 via USP5-mediated deubiquitination, leading to aggravation of ER (endoplasmic reticulum) stress in CRC cells treated with brigatinib, which was an anaplastic lymphoma kinase (ALK) inhibitor and originally approved for ALK-positive NSCLC, thus promoting apoptotic cell death of CRC cells [137]. Moreover, primaquine diphosphate (PD), a known antimalarial drug, was found to be able to inhibit USP1/Uaf1, and then suppress VEGF and histamine-induced vascular permeability [138]. Therefore, it is possible that drugs applied to other diseases are also relevant to deubiquitinase, but further studies are needed to see if they can be effective to USP12 as well.

## Conclusion

In past decades, the protein post-translational modifications have always been researching hotspots, including ubiquitination and deubiquitination, and the balance between them can affect tremendous aspects of biological events. USP is the maximum family of DUBs and has attracted much attention from researchers. USP12, as a member of USPs, possesses numerous physiological functions, involving the influence on cell phenotype, immunity, inflammation, and disease. USP12 can act as a tumor promotor or suppressor, which depends on the specificity of cells or substrates. Moreover, USP12 has a close connection with the tumor immune microenvironment. The relationship of USP12 and PD-L1, TCRs, macrophages, inflammatory microsomes, and various chemokines have been studied and may provide valuable therapeutic strategies in immune treatment. Several signaling pathways are also associated with USP12, including PI3k-Akt, NF-κB, Notch, and p38-MAPK pathways, and USP12 is capable to activate or inhibit these pathways in different diseases.

Generally, USP12 is suggested to be an essential regulator in various biological activities, and new therapies targeting USP12 or related complexes deserve expectation. Hence, the functions and mechanisms behind USP12 deserve further exploration and confirmation in the future.

## Data Availability

Not applicable.

## Abbreviations

PLN: Phospholamban
DSB: DNA double-strand break
DUBs: Deubiquitylases
USPs: Ubiquitin-specific proteases
OTUs: Ovarian tumor-related proteases
UCHs: Ubiquitin C-terminal hydrolases
MJDs: Machado-Joseph disease protein domain proteases
JAMMs: JAB1/MPN/MOV34 motif proteases
MINDY: Motif interacting with ubiquitin-containing novel DUB family
ZUFSP: Zinc finger with UFM1-specific peptidase domain protein
MCPIP: Monocyte chemotactic protein-induced protein
UAF-1: USP1-associated factor 1
pVHL: Von Hippel-Lindau protein with missense point mutations
PHLPP1: PH domain leucine-rich repeat protein phosphatase 1
PHLPPL: PH domain leucine rich repeat protein phosphatase 2
HMGB1: High mobility group box-1
mHTT: HD mutant protein
MDK: Midkine
HUVEC: Human umbilical vein endothelial cell
MMP14: Matrix metalloproteinase 14
MDSCs: Myeloid-derived suppressor cells
IDO1: Indoleamine 2,3 dioxygenase 1
TME: Tumor microenvironment
TAMs: Tumor-associated macrophages
BMDMs: Bone marrow derived macrophages
iNOS: Inducible nitric oxide synthase
TCRs: T cell surface receptors
NLRP3: NOD-like receptor protein 3
IFN: Interferon
CBP: CREB-binding protein
p-STAT1: Phosphorylated STAT1
IFI16: Interferon-γ inducible protein 16
STING: Stimulator of interferon genes
METTL3: Methyltransferase-like 3
Ang-II: Angiotensin II
AR: Androgen receptor
PC: Prostate cancer
MM: Multiple myeloma
ccRCC: Clear cell renal cell carcinoma
HCC: Hepatocellular carcinoma
NSCLC: Non-small cell lung cancer
LUAD: Lung adenocarcinoma
RRM2: Ribonucleotide reductase regulatory subunit M2
HD: Huntington’s disease
ORP8: Oxysterol-binding protein-related protein 8
ALK: Anaplastic lymphoma kinase
PD: Primaquine Diphosphate
